# Screening Strategies for COVID-19 in Patients With Hematologic Malignancies

**DOI:** 10.3389/fonc.2020.01267

**Published:** 2020-07-03

**Authors:** Tarek Assi, Bachar Samra, Laurent Dercle, Elie Rassy, Joseph Kattan, Marwan Ghosn, Roch Houot, Samy Ammari

**Affiliations:** ^1^Department of Hematology and Medical Oncology, Faculty of Medicine, Saint-Joseph University, Beirut, Lebanon; ^2^Department of Leukemia, The University of Texas MD Anderson Cancer Center, Houston, TX, United States; ^3^Radiology Department, Columbia University Medical Center, New York Presbyterian Hospital, New York, NY, United States; ^4^Department of Hematology, CHU de Rennes, Université de Rennes, Rennes, France; ^5^Department of Medical Oncology, Dana-Farber Cancer Institute, Boston, MA, United States; ^6^Radiology Department, Gustave Roussy Cancer Campus, Villejuif, France; ^7^BIOMAPS, UMR1281, INSERM.CEA.CNRS, Université Paris-Saclay, Paris, France

**Keywords:** hematologic malignancies, hematology, COVID-19, coronavirus, screening, polymerase chain reaction, CT scan

## Abstract

COVID-19 has been declared a pandemic by the world health organization. Patients with cancer, and particularly hematologic malignancies may be at higher risk for severe complications due to their malignancy, immune dysregulation, therapy, and associated comorbidities. The oncology community has been proactive in issuing practice guidelines to help optimize management, and limit infection risk and complications from SARS-COV-2. Although hematologic malignancies account for only 10% of all cancers, their management is particularly complex, especially in the time of COVID-19. Screening or early detection of COVID-19 are central for preventative/mitigation strategy, which is the best current strategy in our battle against COVID-19. Herein, we provide an overview of COVID-19 screening strategies and highlight the unique aspects of treating patients with hematologic malignancies.

## Introduction

In December 2019, infection with a novel betacoronavirus, subsequently named SARS-CoV-2, has been reported in a cluster of pneumonia cases in the Wuhan region of China (1). The pathogen showed a rapid spread that led to a global pandemic and a public health emergency of international concern due to the burden on the healthcare system and lack of specific treatments. By April 26th 2020, the COVID-19 outbreak has affected more than 2.8 million people and claimed the lives of more than 190,000 patients around the globe (2). Patients with cancer are regarded as a more vulnerable population because of their immunosuppressive state induced by the malignancy, therapy, and associated comorbidities (3, 4). As a result, despite the limited available data, the oncology community has been pro-actively engaged in issuing guidelines to help clinical practice with the goal of decreasing exposure and complication risks from COVID-19 among patients with cancer. For instance, recommendations have emerged to consider on a case-by-case basis, whenever possible, options of delaying/dose reducing treatment, using lower-intensity therapy, postponing unnecessary radiological evaluations, and prioritizing telemedicine (5–9).

Hematologic malignancies encompass a wide range of neoplasms, including leukemia, lymphoma, multiple myeloma, myelodysplastic syndrome and myeloproliferative neoplasms, which all account for 10% of all malignancies (10). Although some patients present with indolent diseases that may not require immediate therapy, many patients harbor aggressive diseases, often life-threatening, which necessitate rapid intervention with intensive chemotherapy, high dose radiation and/or hematopoietic stem cell transplant (HSCT). Prior studies have shown that patients with hematologic malignancies are at higher risk for severe lower respiratory tract infections, notably due to lymphopenia, neutropenia, hypogammaglobinemia, steroid administration, and graft-versus-host disease, it is unclear how this may be applicable to COVID-19 (11–13).

The triage of patients according to their COVID-19 status (confirmed, high- or low level of suspicion) has been the key measure in limiting in-hospital contaminations (14). The absence of a rapid screening system for assessing COVID-19 status in patients is problematic, and the current recommendations are only based on anecdotal and theoretical evidence extrapolated from the management of other infectious diseases. Herein, we provide an overview of COVID-19 screening strategies and highlight the complex aspects of caring for patients with hematologic malignancies.

## Risk Factors for COVID-19 In Patients With Hematologic Malignancies

Literature on COVID-19 in hematologic malignancies is sparse and mainly derived from case series that are focused on patients with solid tumors. Two cases series from China showed a higher incidence (1 vs. 0.3%), and a higher case-fatality rate (5 vs. 1%) in patients with cancer compared with the overall population (4, 15). However, the relative contribution of cancer or cancer therapy to their infectious risk of COVID-19 is uncertain given that the majority of patients either had a remote history of cancer, were not on active anticancer therapy, and/or had multiple comorbidities. Notably, none of the patients had a hematologic malignancy (4, 15).

Patients with hematologic malignancies might be at higher risk of contracting and experiencing complications from COVID-19, such as hospitalization, intensive care unit (ICU)/invasive ventilation, sepsis, cytokine dysregulation, multiorgan failure and/or death (16). Reasons for this higher risk are multifactorial (Table 1). First, hematologic malignancies are directly tied to an immunocompromised status due to humoral and cellular immune dysfunction (17–20). Second, a large number of therapies employed for hematologic malignancies are highly immunosuppressive, even more than standard chemotherapies used for solid tumors. Examples include myelosuppressive chemotherapy for acute leukemia, conditioning regimens for hematopoietic stem cell transplant (HSCT), prolonged use of steroids, lymphodepleting agents used for chimeric antigen receptor (CAR) T-cell therapy, and radiation therapy. Third, a high proportion of patients with active hematologic malignancies, especially when on active therapy, are constantly exposed to medical facilities and healthcare staff, which puts them at higher exposure risk. This includes frequent travels and visits for outpatient infusional therapies, laboratory checks or transfusions, longer infusions of certain therapies (anti-CD20 antibodies in lymphoma, daratumumab in multiple myeloma), and the need for hospitalization for other therapies (e.g., induction chemotherapy for acute leukemia). Fourth, many hematologic malignancies occur in older patients (median ages in multiple myeloma, CLL, acute myeloid leukemia, follicular lymphoma, and diffuse large B-cell lymphoma are 72, 70, 68, 65, and 64 years, respectively) with multiple coexisting comorbidities (cardiovascular and lung diseases), which are among the highest risk factors for COVID-19-related morbidity and mortality in large Chinese cohorts (4, 15, 21). Fifth, clinical care for patients with hematologic malignancies requires more medical resources (equipment and staff) than for other patients due to the high risk nature of their diseases such as the need for frequent and close monitoring, transfusion burden, high rate of elective and urgent hospital admissions, and crucial need for clinical trial enrollment. Therefore, as clusters of outbreaks can overwhelm the healthcare system capacity, the detrimental effect may be even more pronounced on patients with hematologic malignancies. Sixth and last, many patients with hematologic malignancies may be directly harmed by travel restrictions affecting delivery of crucial therapies such as stem cells and CAR products from unrelated donors (22, 23). On the other hand, a large proportion of patients with hematologic malignancies, especially acute leukemia or transplant candidates/recipients have been adopting social distancing and preventative precautious measures regardless of the COVID-19 crisis, which may have reduced their exposure risk of COVID-19. Moreover, some targeted therapies commonly used in hematologic malignancies, particularly JAK-STAT pathway inhibitors and bruton tyrosine kinase inhibitors (e.g., ibrutinib), have been postulated to have a protective effect in decreasing virus infectivity (24) and abrogating cytokine-mediated lung injury, respectively (25). Clinical trials are ongoing to further investigate the role of such therapies in patients with COVID-19 (NCT04375397, NCT04320277).

**Table 1:** Unique considerations for COVID-19 in patients with hematologic malignancies.

Patient-related factors
Older age patients
Multiple coexisting comorbidities (cardiovascular and lung diseases)
Disease-related factors
Immunocompromised status due to humoral and cellular immune dysfunction
Treatment-related factors
More frequent use of immunosuppressive therapies
Higher exposure risk to medical facilities and staff (frequent travels/medical visits, transfusions, and the need for hospitalization for certain therapies)
System-related factors
Overwhelmed healthcare system and complexity of care
Potential impact of travel restrictions affecting delivery of crucial therapies such as stem cells and CAR T-cells from unrelated donors
Decreased availability or access to crucial and immediate clinical trials (travel restrictions, trials placed on hold, research team understaffing or re-assignments)

*CAR, chimeric antigen receptor*.

## COVID-19 in Patients With Hematologic Malignancies

To our knowledge, at the time of writing this review, only 10 cases of COVID-19 have been published to date in the English literature in patients with hematologic malignancies. Details are summarized in Table 2 (23, 25–27). Data on screening for COVID-19 in hematologic malignancies are limited. A Chinese cross-sectional survey aimed to evaluate the incidence and outcome of COVID-19 among patients with chronic myeloid leukemia (CML) conducted over 1 week in February 2020 (23). Among 392 patients who took the survey, 12 patients were suspected to have COVID-19 infection based on their clinical presentation, but only 2 cases were confirmed using PCR and CT for an incidence of 0.6%. When the authors classified patients according to CML response milestones (according to the 2020 European Leukemia Net guidelines), the incidence of COVID-19 was 0.3% (1 of 299) among patients with optimal response as opposed to 2% (1 of 50) among patients with poor response raising the possibility that good disease control may be related to a lower incidence of COVID-19 (23). These findings must be interpreted with caution given that the relative contribution of CML diagnosis and therapy (imatinib) as a risk-factor may be questionable. The patient with “suboptimal response” to CML therapy was 89 years old with cardiac history, in hematologic remission and only residual molecular disease on Imatinib and succumbed from COVID-19 due to multiorgan failure including myocardial damage. In addition, tyrosine kinase inhibitors (e.g., imatinib) are not myelosuppressive and not known to increase infectious risks especially in the setting of hematologic and cytogenetic remission such as this case.

**Table 2:** Published cases of COVID-19 in patients with hematologic malignancies.

**Disease**	**Demographics/Therapy**	**Presentation**	**Infectious course**	**Treatment**	**Outcome**	**Reference**
MM	60/M/thalidomide maintenance	Hypoxia Chest pain + PCR CT: GGO	Nasal O_2_ support High IL-6	Antibiotics, steroids, and tocilizumab	Recovery	(26)
CLL	39/M/chlorambucil	Fever/respiratory symptoms + PCR CT: GGO	Non-invasive ventilation	IVIG, steroids, nebulized alpha-interferon. Also resumed Reduced dose chlorambucil	Recovery	(27)
WM	***Pt 1:*** 65/M/ibrutinib ***Pt 2:*** 61/M/ibrutinib ***Pt 3:*** 72/F/ibrutinib ***Pt 4:*** 67/F/ibrutinib ***Pt 5:*** 71/M/ibrutinib ***Pt 6:*** 58/M/ibrutinib	***Pt 1–5:*** Fever/respiratory symptoms ***Pt 6:*** Fever/respiratory symptomsCT: GGO	***Pt 1:*** ICU ***Pt 2:*** Not hospitalized ***Pt 3:*** Not hospitalized ***Pt 4:*** Not hospitalized ***Pt 5:*** Not hospitalized ***Pt 6:*** Mechanical ventilation and intensive care unit	***Pt 1:*** HCQ+AZ ***Pt 2–5:*** None ***Pt 6:*** HCQ+AZ+ IVIG, and tocilizumab All pts continued Ibrutinib	***Pt 1:*** Improved ***Pt 2:*** Recovery ***Pt 3:*** Recovery ***Pt 4:*** Recovery ***Pt 5:*** Recovery ***Pt 6:*** Improved	(25)
CML	***Pt 1:*** 89/F/Imatinib ***Pt 2:*** 47/M/HQP1351	***Pt 1:*** Respiratory symptoms + CT: GGO ***Pt 2:*** Fever/respiratory symptoms + PCR CT: negative	***Pt 1:*** Respiratory and renal failure. Myocardial damage ***Pt 2:*** Hospitalized. Mild course	NA	***Pt 1:*** Death 3 days after hospitalization ***Pt 2:*** Recovery	(23)

*MM, multiple myeloma; CLL, chronic lymphocytic leukemia; WM, Waldenstrom macroglobulinemia; CML, chronic myeloid leukemia; M, male; F, female; PCR, polymerase-chain reaction; CT, computed tomography; GGO, ground-glass opacities; IVIG, intravenous immunoglobulin; O_2_, oxygen; HCQ, hydroxychloroquine; AZ, azythromycine*.

The American Society of Hematology Research Collaborative has recently launched a COVID-19 international registry for patients with hematologic malignancies (28). By April 23rd, 2020, there were 64 patients reported to have COVID-19. Underlying malignancies were as follows: acute leukemia (31%), non-Hodgkin lymphoma (22%), chronic lymphocytic leukemias (16%), myeloproliferative neoplasms (16), plasma cell neoplasms (11%), and Hodgkin's lymphoma (6%). Sixty percent of these patients were undergoing active anticancer therapy at the time of COVID-19; among whom 60% were undergoing induction therapy, and 20% were on maintenance/consolidation therapy.

An age-matched comparison of patients with cancer (*n* = 105) and without cancer (*n* = 536) was presented at the American Association for Cancer Research 2020 virtual meeting (29). Multivariable analysis showed that cancer diagnosis was associated with higher rates of death and ICU (intensive care unit) admissions from COVID-19. Patients with hematologic malignancies had the highest severity of symptoms (6 out 9 patients [66.67%]) high risk of ICU admission or use of invasive mechanical ventilation [4 and 2 out 9 patients, respectively [44.4 and 22.2%] and death rate (3 out 9 patients [33%]). Although authors reported that 4 out of the 9 patients with hematologic malignancies had severe immunosuppression, no information was provided on disease status or treatment details. Therefore, the small sample number and lack of detailed clinical data on patients with hematologic malignancies limit generalizability of this observation.

## Screening Strategies

The COVID-19 pandemic has added another major burden to cancer care of all specialty's units, especially hematology and HSCT units. Due to limited testing and screening, little information is known about the incidence of asymptomatic carriers, who maybe as contagious as symptomatic patients (30–32). Efforts have been made to triage COVID-19 patients to separate units in order to avoid inpatient cross-contamination to other patients, thus, highlighting the importance of screening strategies. Additional testing strategies have been rapidly developed to better identify patients with COVID-19. Since SARS-CoV-2 is an RNA-virus, reverse transcriptase-polymerase chain reaction (RT-PCR) is the obvious diagnostic test to confirm virus shedding (33). However, the sub-optimal sensitivity has limited its reliability in clinical practice. The test can be obtained from nasopharyngeal swabs or sputum samples, and results are generally reported within 4–48 h (34). Nasopharyngeal swabs may lack sensitivity after the first week of COVID-19 infection due to the natural history of the disease. The virus was shown to transiently infect the upper respiratory system until it spreads more toward the lungs. This is extrapolated from data showing a higher rate of RT-PCR positivity >90% on days 1–3 of illness, vs. <80% at day 6, and vs. <50% after day 14 (35). False negatives can also be attributed to the poor quality of the specimen and type of specimen obtained (36). Another appealing screening strategy is serologic testing of SARS-CoV-2 antibodies to identify persons that are potentially “immune” and thus at minimal risk of contagion or complications, both at individual and public health levels (37). This also has important therapeutic implications by identifying potential candidates to donate blood for the preparation of convalescent plasma, an investigational product in the management of severely ill COVID-19 patients (38).

Due to limitations associated with PCR testing, including limited testing capacity, high false-negative rates, and delays in having the results, chest imaging has emerged as a potent diagnostic tool for COVID-19. Chest radiographs have a low sensitivity in early or mild disease and therefore, are not ideal screening tools. In one study, twenty percent of patients had normal chest radiographs at any point during the course of their illness (39). In contrast, chest computed tomography (CT) scan is more sensitive, especially in the presence of typical findings, such as bilateral, peripheral patchy ground-glass opacities, predominantly in the lower lobes (33, 40, 41). Although the American College of Radiology recommends that chest CT scan be reserved for symptomatic patients with suspected COVID-19-related complications and discourages its use for screening purposes, these guidelines may not be applicable for patients with cancer, especially hematologic malignancies undergoing immunosuppressive therapies (42). Since many radiological findings may be seen with other etiologies such as opportunistic infections or drug-induced pneumonitis, the role of radiologists is primordial in confirming diagnosis of COVID-19 (43). Vascular thickening, and peripheral distribution seem to be more characteristic of COVID-19 than other viral infections (44). The concordance between PCR and CT scan has been addressed in a Chinese cohort from the Wuhan region of 1,014 patients with suspected COVID-19. Ninety-seven percent of patients had positive CT scans. However, important observations were notable. Initially, CT scan was more sensitive in detecting early infections (88%, compared with 59% with PCR). Moreover, CT scan was better in assessing recovery; 42% had signs of radiological improvement before RT-PCR turned negative (33). Therefore, CT scan may have a higher yield than PCR for diagnosis and monitoring. Similar findings were shown by Fang et al. (40) (chest CT sensitivity of 98 vs. 71% with PCR; *p* < 0.001). One meta-analysis showed a positive predictive value reaching 90.4% for chest CT scan. Taken together, these findings support the superiority of CT over PCR for detection and screening of COVID-19 especially in endemic areas and for higher-risk patients such as those with hematologic malignancies. Nonetheless, many challenges remain before implementation to screen for asymptomatic carriers (Table 3) (45). Artificial intelligence (AI) techniques may increase the sensitivity and specificity of imaging tools to screen for COVID-19 infections (46). Deep learning techniques have been successfully used to differentiate bacterial and viral infections with specific lung injuries, suggesting that it could be a promising tool for the diagnosis and screening of COVID-19 (47, 48). AI can improve image acquisition through automation of the scanning procedure and reshaping of the workflow, which would limit the human-to-human contact and avoid virus transmission. These techniques can also increase the accuracy of the COVID-19 diagnosis through the delineation of suspected infection on chest CT scan and radiography with different segmentation methods (49). Deep learning tools for the detection of COVID-19 infections are currently being developed with promising results such as the COVID-19 detection neural network (COVNet) (50).

**Table 3:** Screening strategies for COVID-19 patients.

**Screening Test**	**Advantages**	**Limitations**
RT-PCR	Ease of testing Standardized	High false negative rates Limited accessibility Delay in test result
Chest-X Ray	Low cost Easy access Rapid result	Low sensitivity Radiation exposure
Chest CT scan	High sensitivity Great tool for AI techniques Rapid result	Low specificity Radiation exposure
Antibody serology	identification of immune response Potential therapeutic implication (plasma donors)	Not standardized Limited access

## Published Guidelines for COVID-19 Screening

Despite the paucity of current available data, several medical societies have provided clinical practice guidelines for management of patients with hematologic malignancies in order to optimize therapy and limit infection risk and complications (9, 51). Consensus recommendations have been based on extrapolated information from other coronavirus epidemics and expert opinions based on educated assumptions. Social distancing, quarantine measures, clear guidance on the importance of hand hygiene, and avoiding high-risk exposures are recommended to halt the virus transmission (52). Preventative strategies also include screening for COVID-19 symptoms or fever at the hospital entrance for all patients, caregiver/visitors, and healthcare workers (53). Serial RT-PCR screening of healthcare workers, once every week or every 2 weeks can be another proposed measure for infection control (54). Initial evaluation of patients with hematologic malignancies or undergoing hematopoietic cell transplantation should at least include clinical history and examination, and laboratory and radiological evaluation to document active respiratory viral infections (55, 56). In patients with acute myeloid leukemia, up to 50–75% of patients present with fever and thus are at risk of delayed or missed diagnosis (57, 58). The European Hematology Association recommends testing for COVID-19 with RT-PCR in all newly diagnosed patients with acute and chronic leukemia undergoing intensive chemotherapies and before every cycle of therapy. No recommendations regarding the screening role of chest CT scan have been issued (5). According to the American Society of Hematology guidelines, screening of patients with chronic lymphocytic leukemias presenting with mild symptoms depends on the availability of testing and the need to isolate those who test positive (59). Regarding multiple myeloma, it is generally recommended to screen all newly diagnosed patients for COVID-19 in the inpatient and outpatient settings (60, 61). Screening is widely used and strongly recommended before autologous and allogeneic HSCT (6, 62).

According to the European Society for Blood and Marrow Transplantation guidelines, COVID-19 should be ruled-out in all patients, including asymptomatic ones, before undergoing conditioning chemotherapy (62). Additionally, patients planned to receive CAR-T cell therapy or HSCT should abide by home isolation for at least 2 weeks before lymphodepleting or conditioning chemotherapy. In case of close exposure to a patient with confirmed COVID-19, any elective therapy should be deferred for at least 2–3 weeks before undergoing a new RT-PCR screening to confirm negativity (62, 63). As for donors, COVID-19 positive patients should be excluded while those in contact should be isolated for 4 weeks unless the patient is asymptomatic and the procedure is urgent, then it can be considered after negative testing.

Screening measures in the pre-CAR T cell phase should include screening for symptoms for all patients before apheresis and CAR T cell infusion. RT-PCR testing is considered within 48–72 h before apheresis and recommended within 48–72 h before lymphodepleting chemotherapy and 7 days within CAR T cell infusion. A repeat evaluation of RT-PCR within 72 h of CAR T cell infusion to detect interim COVID-19 infection and perform serologic testing for COVID-19 seroconversion once available (22, 64). That being said, stem cell and CAR-T cell recipient patients residing in high-endemic areas or who have been in contact with suspected COVID-19 patients should undergo RT-PCR testing while those who tested positive should undergo a CT scan and oxygen need evaluation (62). The American Society for Transplantation and Cellular Therapy recommends that asymptomatic patients whose cellular therapy cannot be delayed should be tested within 72 h before their admission or initiation of the conditioning regimen. Also, asymptomatic allogenic donors should be screened 72 h before collection while autologous donors with chemo-mobilization should be screened 72 h before chemotherapy and cell collection and those on GCSF and plerixafor on the day of GCSF start, respectively (6).

Last, blood transfusions should be carefully monitored during this outbreak. Blood donors should be screened for any suspicious signs or close exposure to COVID-19 present in at least the past 2 weeks (65). The WHO has issued specific guidelines on maintenance and safety of blood products supply during the COVID-19 pandemic (66).

## Conclusion

**Figure 1:**
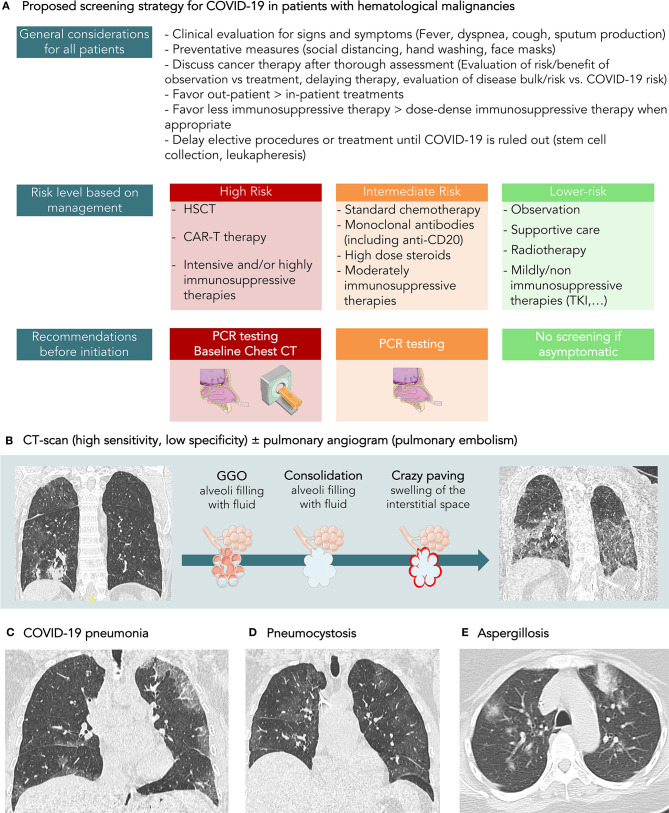
**(A)** Proposed screening strategy for COVID-19 in patients with hematological malignancies. **(B)** CT-scan is a sensitive tool, but its specificity for the diagnosis of COVID-19 is around 37%, according to recent meta-analysis. It has not been evaluated in patients with hematological malignancies but could be even lower due to a higher rate of infection caused by immune suppression. **(C)** COVID-19 pneumonia in a 58-year-old patient with diffuse B lymphoma treated with R ACVBP. Symptoms were cough and fever. SARS-CoV-2 rt-PCR was positive. On CT-scan, the CT score CORADS was 5. The axial CT image showed ordinary COVID-19 pneumonia with multiple regions of subpleural GGO (Ground Glass opacity) with superimposed inter and intralobular septal thickening. **(D)** Pneumocystis in a 58-year-old patient followed for acute leukemia. The clinical exam revealed a cough and fever. The patient was lymphopenic. CT-scan showed diffused ground-glass opacities. SARS-CoV-2 rt-PCR was negative. The final diagnosis was pneumocystis. **(E)** Diffuse pulmonary condensation in a 55-year-old patient with AML in febrile aplasia. SARS-CoV-2 rt-PCR was negative. A positive aspergillus antigenemia was confirmed by culture of bronchoalveolar lavage fluid.

Patients with hematologic malignancies are thought to be particularly vulnerable for severe illness from COVID-19. The outbreak has added a large burden to the complexity of cancer care in hematologic malignancies on many aspects. Balancing the management of COVID-19 and its complications and management of cancer is particularly challenging in patients with hematologic malignancies, especially when treating with curative intent, which is often the case in many blood cancers. In the context of limited available scientific data in a such young and rapidly evolving field, physicians are left for now with their clinical judgement, educated guesses and consensual expert opinions (Figure 1). Until the advent of effective anti-viral therapy or vaccines, preventative behavioral strategy remains our best model to help protect our patients. Screening for asymptomatic carriers and immune persons has a central role in optimizing mitigation strategy to maintain safety at individual and public health levels. Despite the availability of RT-PCR, antibody serology, and CT scan as complementary diagnostic tools, several limitations exist in implementing a cost-effective screening strategy that can be applicable on a large scale. A tiered approach targeting patients at pre-defined higher risk (pre-conditioning or other intensive chemotherapy regimens, before prolonged use of steroids or anti-CD20 antibodies etc.) may be a better way and warrants more exploration. Despite the numerous difficulties during the pandemic, the oncology community has been very adaptive in adjusting to the new challenges. In addition, scientific and technological progress is evolving rapidly, which gives hope to the oncology, and international communities.

## Data Availability Statement

The original contributions presented in the study are included in the article/supplementary material, further inquiries can be directed to the corresponding author/s.

## Author Contributions

All authors contributed equally to the manuscript writing and review.

## Conflict of Interest

The authors declare that the research was conducted in the absence of any commercial or financial relationships that could be construed as a potential conflict of interest.
